# 1,550-nm photonic crystal surface-emitting laser diode fabricated by single deep air-hole etch

**DOI:** 10.1515/nanoph-2024-0760

**Published:** 2025-02-14

**Authors:** Myeongeun Kim, Ye-Seong Song, Lakjong Jeong, Tae-Yun Lee, Hyo Seok Choi, In Kim, Myungjae Lee, Heonsu Jeon

**Affiliations:** Department of Physics and Astronomy, 26725 Seoul National University, Seoul 08826, Republic of Korea; Inter-University Semiconductor Research Centre, 26725 Seoul National University, Seoul 08826, Republic of Korea; OE Solutions, Gwangju 61080, Republic of Korea; Department of Materials Science and Engineering, 26725 Seoul National University, Seoul 08826, Republic of Korea; Research Institute of Advanced Materials, 26725 Seoul National University, Seoul 08826, Republic of Korea; Institute of Applied Physics, 26725 Seoul National University, Seoul 08826, Republic of Korea

**Keywords:** photonic crystal, surface-emitting laser, laser diode, band-edge mode, deep air hole, dry-etch

## Abstract

Photonic crystal surface-emitting lasers (PCSELs) are promising light sources with numerous advantages, including vertical emission, single-mode operation, and high output power. However, the fabrication of PCSEL devices requires advanced techniques, such as wafer bonding or epitaxial regrowth, to form a photonic crystal (PhC) structure close to the central waveguide layer. This process is not only complicated but also necessitates multiple semiconductor epitaxies, which reduces fabrication yield and increases manufacturing costs. In this study, we introduce a simpler method for fabricating PCSELs that requires only a single dry-etch run on any standard edge-emitting laser diode epistructure. The key challenge of creating an array of PhC air holes deep enough to reach the waveguide layer is addressed through high-temperature, high-plasma-density dry etching. PCSEL devices fabricated using this method lased in single mode at a threshold current density as low as ∼0.8 kA/cm^2^, which is comparable to or better than previously demonstrated devices. Our results offer a cost-effective, high-yield approach to PCSEL fabrication.

## 1 Introduction

Since their initial demonstration using optical excitation [1], photonic crystal (PhC) lasers – utilizing a full photonic band-gap – have been anticipated as next-generation light sources due to their small footprint and low power consumption. However, the development of PhC lasers has been hindered by difficulties in converting them into electrically drivable laser diodes (LDs). Although a few proof-of-concept devices have been demonstrated [2], [3], they are applicable only for specific kinds of cavity modes. PhC LDs with lateral p–n junctions [4], [5], on the other hand, require complex ion implantation techniques to reconstruct lateral doping profiles, making them less attractive for practical use. Therefore, more ingenious PhC LD platforms are still to come.

Meanwhile, Noda’s group developed electrically operated photonic crystal surface-emitting lasers (PCSELs) [6], [7] that only require a partial band-gap, where a two-dimensional (2D) PhC structure composed of shallow air holes is placed near a core waveguide layer containing optically active multiple-quantum wells (MQWs). PCSELs, characterized by vertical laser emission and high output power in single mode, have numerous application potentials, such as light-detection-and-ranging (LiDAR) [8], [9], high-speed optical data transmission [10], laser material processing [11], and solid-state laser pumping [12]. In terms of operation principles, PCSELs are a special kind of band-edge lasers that utilize a Γ-point band-edge mode. The in-plane photon momentum *k*_||_ is zero at the Brillouin zone center, allowing for vertical light emission, thus classifying these devices as surface-emitting lasers (SELs). PCSELs operate in a single mode regardless of lateral size, allowing for power scaling without triggering higher-order transverse lasing modes, a key advantage over vertical-cavity SELs. The development of PCSELs has been highly successful, achieving numerous milestones, including high output power (up to 50 W in continuous-wave [13] and 80 W in pulsed mode [14]), polarization control [15], [16], narrow beam divergence [17], on-chip beam steering [18], [19], [20], operation at various wavelengths (visible [21], near-infrared [13], [14], telecommunications [6], [22], [23], [24], [25], [26], [27], and terahertz [28]), and commercialization. Despite their successful development, current PCSELs require sophisticated fabrication processes, such as wafer fusion [6], [7], [15], [16] or epitaxial regrowth [13], [14], [17], [19], [20], [21], [22], [23], [24], [25], [26], [27], to insert a 2D PhC structure close to the waveguide layer. This complexity increases production costs and reduces fabrication yield.

In this study, we present 1,550-nm PCSEL devices that greatly simplify the fabrication process and eliminate the need for dedicated epitaxial regrowth. Starting with a standard edge-emitting LD epistructure, only a high-aspect-ratio air-hole array needs to be etched through the cladding layer on one side of the p–n junction. Despite the simplicity of the device structure and fabrication process, the performance of these PCSELs is comparable to that of existing devices produced through far more complex methods.

## 2 Design of device structure

**Figure 1:**
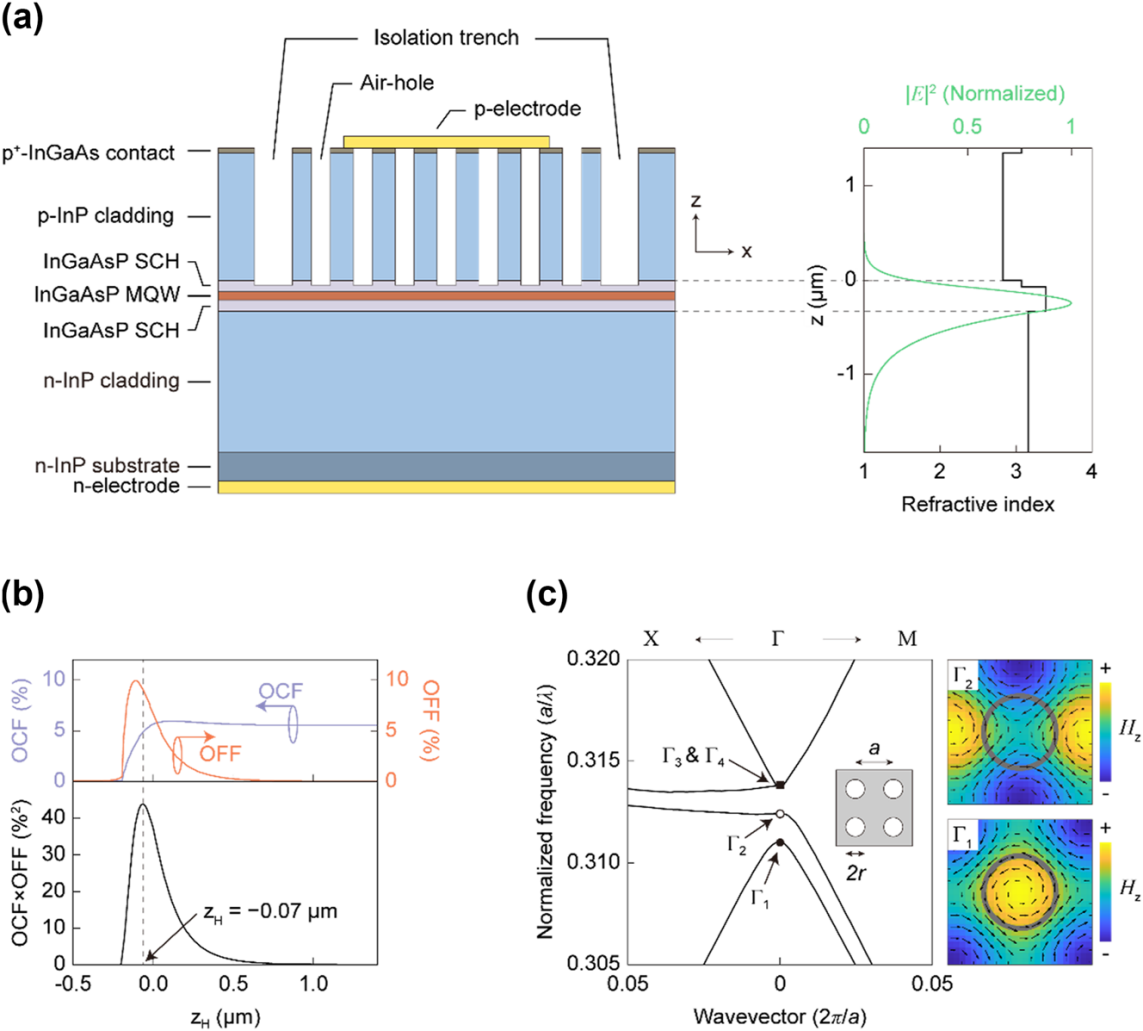
PCSEL structure. (a) Cross-sectional schematic of the PCSEL device (left) and vertical guided mode profile calculated for the optimum PCSEL structure (right) – along with the effective refractive index profile. (b) The OCF and OFF (upper) and the figure-of-merit OCF × OFF (lower), both plotted as a function of the position of the air-hole bottom *z*_H_. The dashed vertical line indicates the optimum air-hole depth: *z*_H_ ≈ −0.07 μm. (c) Photonic band structure calculated for the optimum PCSEL structure. Shown in the right are the lateral electric field (*E*_||_) distributions (in arrows) and the vertical magnetic field (*H*_*z*_) profiles (in color) for the Γ_1_ and Γ_2_ band-edge modes.

The base wafer used in this study was a standard InP-based Fabry–Pérot LD epistructure configured as a separate-confinement heterostructure (SCH) with five InGaAsP MQWs, designed to emit at 1,550 nm (Supplementary Information S1). The SCH epistructure was designed to support only the fundamental transverse electric (TE) waveguide mode. Any similar LD epistructure with comparable specifications could also be used. A 2D square-lattice PhC array of deep circular air holes was etched through the upper p-cladding layer, as shown in the schematic of the device structure in the left panel of Figure 1(a). The nominal lattice constant and air-hole radius of the PhC structure were set to *a* = 485 nm and *r*/*a* = 0.25, respectively.

For structural optimization, the fundamental TE-guided optical mode profile was calculated as a function of the air-hole depth, assuming that the effective refractive index of the etched part of the cladding layer was the areal average of the refractive indices of InP and air. Two key parameters were extracted from the calculated modal profiles: the optical confinement factor (OCF) and the optical feedback factor (OFF), which represent the overlap integrals of the TE-guided mode with the MQWs and the section where the air holes are formed, respectively. Calculation examples for a few different air-hole depths can be found in Supplementary Information S2. OCF represents the attainable modal gain, while OFF indicates the strength of the PhC effects. The upper panel of Figure 1(b) shows the OCF and OFF as functions of the vertical position of the bottom of the etched air holes, *z*_H_. The origin of the *z*-coordinate is the interface between the p-cladding and SCH layers; hence, *z*_H_ = 0 means the entire p-cladding layer is etched. As the air-hole depth increases (or *z*_H_ > 0 decreases), the OCF remains fairly constant and then decreases once the air holes reach the SCH layer. Meanwhile, the OFF starts at zero and rises when the air holes approach the waveguide layer, but it falls off sharply once *z*_H_ becomes negative. These results can be qualitatively explained as follows: when *z*_H_ > 0, the SCH layer remains unaffected, maintaining its waveguiding properties, while the PhC effects increase as *z*_H_ approaches 0. However, when *z*_H_ < 0, the effective refractive index of the SCH layer decreases rapidly, eventually becoming lower than that of the n-cladding layer, eliminating support for any guided mode and nullifying both the OCF and OFF.

To determine the optimal air-hole depth for the PCSEL, the OCF and OFF parameters are multiplied to form a figure of merit (FOM) [29]. As shown in the bottom panel of Figure 1(b), the FOM reaches its maximum at *z*_H_ ≈ −0.07 μm, where OCF ≈ 4.8 % and OFF ≈ 9.1 %. The right panel of Figure 1(a) displays the guided mode profile for the PCSEL structure with the highest FOM, alongside the effective refractive index profile. The modal peak is only slightly offset from the MQWs, though the guided mode becomes asymmetric due to the air holes in the top p-cladding layer. Achieving the optimum condition of *z*_H_ ≈ −0.07 μm requires slightly overetching the air holes into the SCH layer. To secure fabrication margin, however, we chose a somewhat relaxed FOM of 35(%^2^) – not the maximum FOM of 44(%^2^), which results in the range for etch depth: −0.12 μm < *z*_H_ < 0.01 μm. Thus, we targeted *z*_H_ ≈ 0 in the deep air-hole etching, which offers the highest FOM achievable without etching the MQW-containing waveguide layer. Importantly, the optimal air-hole depth for the proposed PCSEL can be determined for any standard LD epistructure, unlike conventional PCSELs, which require specific epistructures designed for wafer bonding or regrowth.

Figure 1(c) illustrates the photonic band structure of the TE-polarized fundamental waveguide mode in the resulting PCSEL structure, calculated using the finite-difference time-domain (FDTD) method. Four band-edge points, including the degenerate Γ_3_ and Γ_4_, are clearly resolved at the Brillouin zone center. The PCSEL is designed so that the MQW emission at *λ* ≈ 1,550 nm aligns with Γ_1_ or Γ_2_. Shown in the right side of Figure 1(c) are the lateral electric field distributions (*E*_||_, arrows) and vertical magnetic field profiles (*H*_*z*_, colors) for the two Γ_1_ and Γ_2_ band-edge modes. Based on the even symmetry of the *H*_*z*_ field profiles, both Γ_1_ and Γ_2_ band-edge modes are *bound states in the continuum* (BIC) [30], which are known for their high-quality factors, making them desirable for lasing action. On the contrary, Γ_3_ and Γ_4_ possess the odd symmetry in their modal profiles (Supplementary Information S3), thus belonging to non-BIC modes; this is the reason that Γ_1_ or Γ_2_, not Γ_3_ or Γ_4_, is targeted in our device design. It is also worth noting that Γ_1_ and Γ_2_ belong to dielectric bands, for which electric field profiles are concentrated where the p-cladding layer remains intact (no air hole), thus holes being injected and optical gain being provided. This is not the case for Γ_3_ and Γ_4_, which belong to air bands.

## 3 Device fabrication

**Figure 2:**
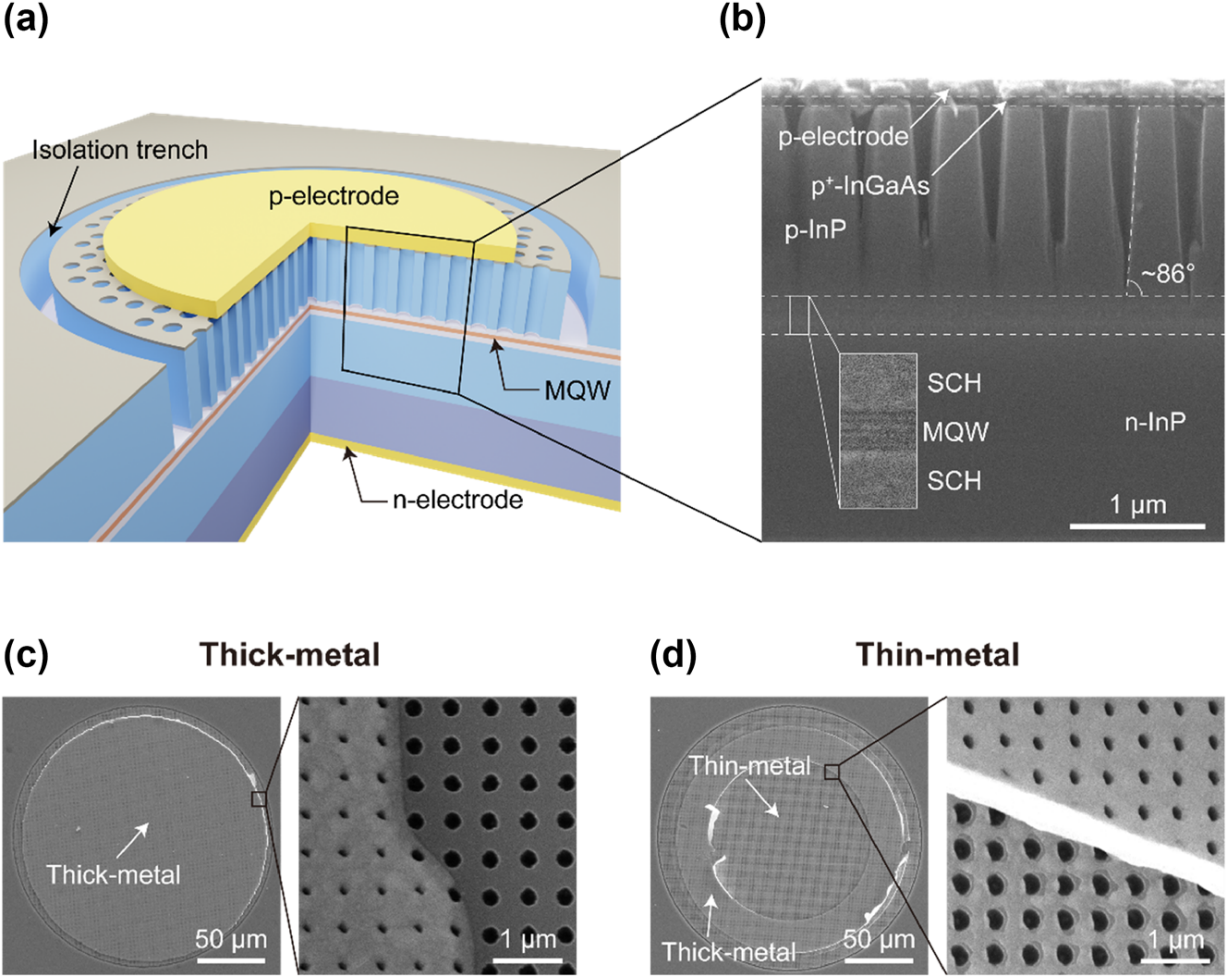
PCSEL devices. (a) Perspective sketch of a fully fabricated PCSEL device. (b) Cross-sectional SEM image of a PCSEL device. The inset shows details of the MQW section. (c) SEM image (top-view) of a PCSEL device fabricated in the thick-metal contact scheme. The enlarged image in the right shows the boundary between the thick p-metal contact and the bare PhC region. (d) SEM image (top-view) of a PCSEL device fabricated in the thin-metal contact scheme. The enlarged image in the right shows the boundary between the thin p-metal contact and the thick metal pad.

The overall configuration of the PCSEL device after fabrication completion is illustrated in Figure 2(a). The fabrication process began with the sequential deposition of a 500-nm-thick SiO_2_ layer and a 20-nm-thick Cr layer. An unusually thick SiO_2_ hard mask was used to enable deep air-hole etching through the p^+^-InGaAs contact layer and p-InP upper cladding layer. The Cr layer served as an etch mask for patterning the thick SiO_2_ layer. The circular, square-lattice PhC pattern was created using electron-beam lithography and transferred, in sequence, to the Cr and SiO_2_ hard masks, and finally to the semiconductor epistructure via dry etching. The diameter of the circular PhC pattern was set at *ϕ* = 200 μm. As described previously, the nominal lattice constant and air-hole radius were *a* = 485 nm and *r*/*a* = 0.25, respectively. Standard reactive-ion etching (RIE) was used for the Cr and SiO_2_ hard masks, while particular care was taken in etching the submicron-sized, deep air holes through the ∼1.35-μm-thick p-InP cladding layer, a process completed with inductively coupled plasma (ICP) RIE at an elevated temperature in a gas mixture of Cl_2_–O_2_–Ar. Further details on the ICP-RIE process for deep InP air holes with high aspect ratios are included in the Methods section.

Figure 2(b) presents a cross-sectional scanning electron microscopy (SEM) image of a typical PCSEL device, demonstrating that deep air holes were successfully etched through both the p^+^-InGaAs contact layer and the full depth of the p-InP cladding layer, achieving an aspect ratio of approximately 2*r*:*d* ≈ 1:5.4, where *d* is the etched air-hole depth. The air-hole sidewalls were slightly angled rather than perfectly vertical, with an estimated sidewall angle of ∼86°. Although not visible in the SEM image, a circular trench (1 μm in width) for device isolation was simultaneously etched around the PhC pattern area during the deep air-hole etch, as illustrated in Figure 2(a). We acknowledge that selective-area epitaxy could be an alternative method to fabricate high aspect ratio nanostructures with smooth and vertical sidewalls [31], [32].

Following etching, a circular p-metal contact (*ϕ* = 180 μm) was deposited by metal lift-off within the PhC air-hole region. To prevent metal from accumulating inside the etched air holes, which could lead to electrical issues (such as higher contact resistance, leakage current, or even short-circuit), the contact metals were deposited at an oblique angle of 45°. Two p-metal contact schemes were applied to the PCSEL devices: a thick-metal contact (35 nm Ti and 140 nm Au) and a thin-metal contact (3.5 nm Ti and 35 nm Au), the latter allowing direct observation of laser emission through the thin metal contact. The 35-nm-thick Au layer in the thin-metal contact, which is already thicker than the Au skin depth (*δ* ≈ 20 nm at *λ* = 1,550 nm), is a compromise between optical transmission and lateral electrical resistance. Figure 2(c) and (d) shows SEM images of devices with thick and thin metal contacts, respectively. An annular thick metal pad was added to the thin metal contact by metal lift-off to facilitate contact with the electrical probe tip. For the n-metal contact, 50-nm-thick Ti and 200-nm-thick Au were deposited on the back side of the wafer, and the sample was annealed to establish ohmic contacts on both p- and n-metal sides.

## 4 Device characterizations

**Figure 3:**
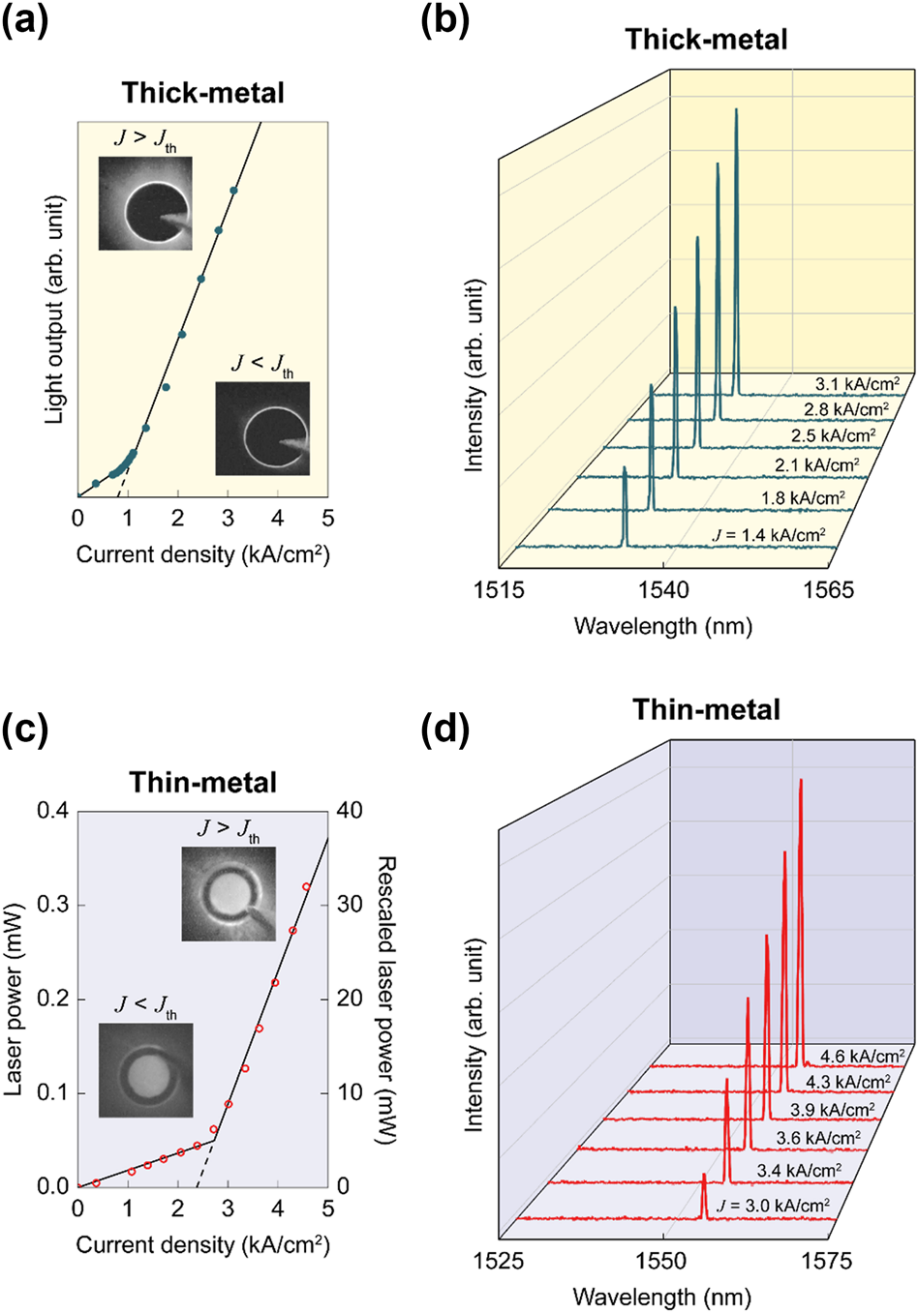
PCSEL performance. (a) Relationship between light output and injected current density of a PCSEL device fabricated in the thick-metal contact scheme. The insets are emission images taken below and above laser threshold. (b) Emission spectra taken at a few representative current injection levels. Each spectrum corresponds to the data point in (a). (c) Relationship between laser power and injected current density of a PCSEL device fabricated in the thin-metal contact scheme. Again, the insets show the emission images below and above laser threshold. (d) Emission spectra taken at a few injection levels. Each spectrum corresponds to the data point in (c).

The fabricated PCSEL devices were tested under pulsed current injection at frequencies ranging from 1 to 10 kHz, with a fixed pulse duration of 1 μs. To simplify measurements at this developmental stage, testing was conducted on a probe station without die mounting or wire bonding. A metal probe tip was placed directly on either the circular metal contact pad in the thick-metal device or the annular metal pad in the thin-metal device. Figure 3(a) depicts the relationship between light output and injected current density (*L*–*J*) for a thick-metal PCSEL device. The light output is plotted on an arbitrary scale, as the majority of laser emission was blocked by the thick contact metal, with only edge-leaked light detectable. Nevertheless, a clear slope change was observed at a threshold current density *J*_th_ ≈ 0.8 kA/cm^2^, indicating a threshold comparable to or lower than those of PCSELs reported in the literature [22], [23], [24], [25], [26], [27], suggesting a high quality of fabrication and device performance. The insets show infrared camera images below and above the threshold: below the threshold, a ring-shaped emission along the isolation trench was observed, while above the threshold, an intense, solar-corona-like emission appeared outside the trench, indicating stimulated emission. Figure 3(b) shows the spectral evolution of the PCSEL device above the threshold, confirming single-mode lasing. It is unfortunate that the data do not clarify which band-edge mode, either Γ_1_ or Γ_2_, is responsible for lasing. The issue on the modal uncertainty in square-lattice PCSELs has been around but resolved – rather than debated – through modal discrimination and controls. The idea is to modify the PhC structure, either changing the air-hole shape [33], [34], [35] or employing a multiple-lattice [13], [17], [36], which in turn results in higher output power and narrower divergence angle with a stable single-lobed far-field pattern.

As described earlier, PCSEL devices with semi-transparent thin metal contacts were also prepared to quantitatively assess laser power. Figure 3(c) and (d) shows the *L*–*J* relationship and emission spectra measured from a typical thin-metal device, respectively. The overall performance characteristics are similar to those of the thick-metal device, except that the threshold current density (*J*_th_ ≈ 2.4 kA/cm^2^) is now three times higher, due to inefficient and nonuniform lateral current spreading through the thin contact metal layer. However, as intended, the entire device area, except for the annular thick-metal contact region, lit up, as shown in the inset images in Figure 3(c). This contrasts with the thick-metal device, shown in the insets of Figure 3(a), where there is no light emission in the central area. Laser output power up to *P* ≈ 0.32 mW was measured through the thin-metal contact at an injection level of *J* ≈ 4.6 kA/cm^2^ (or *I* ≈ 1.16 A). The low laser power is primarily due to the low light transmission through the metal contact layer. Assuming that the Ti and Au layers constituting the thin-metal contact are perfectly uniform and possess the material properties of their bulk counterparts, the estimated transmittance at *λ* = 1,550 nm is still low, at *T* ≈ 0.03 (Supplementary Information S4). The reduced emission area, caused by the thick annular contact pad, also contributes to the low laser output. Taking these factors into account, the actual laser output power can be multiplied by a factor of ∼100, and the rescaled laser power based on this adjustment is shown on the right ordinate of Figure 3(c). The use of a more efficient semi-transparent p-metal contact, such as indium tin oxide [29], [37], or flip-chip bonding with an open n-metal contact [13], [21] should enable better light extraction and thus a more accurate assessment of PCSEL devices.

**Figure 4:**
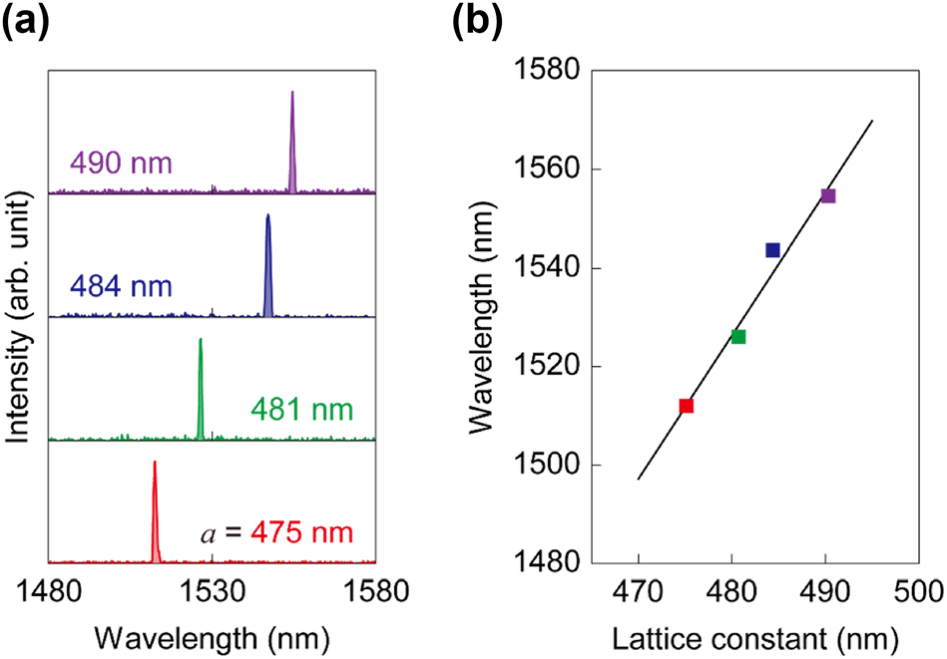
PCSEL wavelength tuning. (a) Emission spectra taken from four PCSEL devices with different lattice constants. (b) Single-mode emission wavelengths as a function of lattice constant for the four PCSEL devices shown in (a), along with a linear fit.

The single-mode lasing wavelength of PCSELs can be tuned by simply adjusting the PhC lattice constant. To demonstrate this wavelength tunability, a set of PCSELs with different lattice constants was fabricated and tested. Figure 4(a) shows the emission spectra of the samples, where it is evident that the single-mode lasing peak redshifts as the lattice constant increases. The lasing wavelengths obtained experimentally from Figure 4(a) are plotted in Figure 4(b), along with a linear fit that indicates a wavelength shift rate of d*λ*/d*a* ≈ 2.9 nm/nm. To confirm this theoretically, FDTD simulations were performed to trace the spectral positions of the Γ-point band-edge modes. Both the Γ_1_ and Γ_2_ band-edge modes exhibited nearly identical wavelength shift rates of d*λ*/d*a* ≈ 3.1 nm/nm, which is reasonably close to the experimental value. This strongly supports the idea that the lasing originates from the Γ-point band-edge mode.

## 5 Conclusions

A novel nanofabrication technique has been developed to etch deep air holes with high aspect ratios into InP-based semiconductors, greatly simplifying the fabrication of 1,550 nm PCSELs. Unlike conventional PCSEL fabrication methods that require wafer fusion or epitaxial regrowth, this approach involves a single-step etching process. By etching an array of deep air holes through the top cladding layer, any conventional edge-emitting LD epistructure can be transformed into PCSEL devices, eliminating the need for more complex fabrication techniques. The key challenge – creating deep InP air holes – was successfully overcome using high-temperature ICP-RIE with a Cl_2_–O_2_–Ar gas mixture. A high substrate temperature facilitated the removal of etch by-products from the deep air holes, enhancing subsequent etching by reactive, high-density plasma. Under pulsed current injection, our PCSEL devices achieved single-mode lasing with a threshold current density as low as *J*_th_ ≈ 0.8 kA/cm^2^, demonstrating the high quality of these devices. The PCSEL nature of the single-mode lasing was maintained regardless of the injection current level. The measured wavelength shift as a function of lattice constant aligned well with band structure calculations, confirming that lasing originates from the Γ-point band-edge mode. Looking ahead, flip-chip bonding should enable more detailed performance assessments and allow for the continuous-wave operation of these PCSEL devices.

## 6 Methods

### 6.1 Device fabrication

A 500-nm-thick silicon dioxide hard mask was deposited onto a standard InGaAsP MQW LD epistructure using plasma-enhanced chemical vapor deposition at 350 °C, followed by the deposition of a 20-nm-thick chromium layer via e-gun evaporation. A positive electron beam resist (ZEP520A, Zeon) was spin-coated onto the Cr hard mask to a nominal thickness of 350 nm. PCSEL patterns were created using electron-beam lithography (JBX-6300FS, JEOL). After resist development, the patterns were sequentially transferred to the Cr and SiO_2_ hard masks by reactive-ion etching (RIE 80 Plus, Oxford Instruments) and then to the InP epistructure using ICP-RIE (PlasmaPro 100 Cobra 300, Oxford Instruments) at an elevated temperature of 200 °C. The gas mixture ratio during the high-temperature ICP-RIE was Cl_2_:O_2_:Ar = 3:1:12. After completing the dry etching, the remaining SiO_2_ hard mask was removed via wet chemical etching. A circular p-metal contact was then formed within the PhC area using microphotolithography, e-gun evaporation (at an oblique angle of 45°), and metal lift-off. The p-metal contact consisted of 35-nm-thick Ti and 140-nm-thick Au in the thick-metal scheme, and 3.5-nm-thick Ti and 35-nm-thick Au in the thin-metal scheme. In the thin-metal scheme, an additional round of thick metal deposition and liftoff was performed to create a ring-shaped metal pad (24 μm in width). Finally, thermal annealing at 350 °C for 30 s completed the device fabrication.

### 6.2 Electrical injection and optical measurements

The fabricated PCSEL LD devices were electrically driven under pulsed conditions (repetition rate of 1–10 kHz, pulse width of 1 μs) using a short-pulse generator (AV-1015-B; Avtech Electrosystems). The devices were tested on a probe station equipped with tungsten probe tips, without requiring sophisticated packaging processes such as die separation/attachment or wire bonding. The injection current and pulse shape were monitored by measuring the voltage drop across a 3 Ω dummy resistor connected in series with the device. Laser emission was collected using a microscope objective lens with a long working distance and analyzed with an optical spectrum analyzer (MS9740A, Anritsu). Device operation was captured through the same objective lens using an infrared video camera (C2741-03, Hamamatsu). Vertical laser output from the thin-metal device was measured directly from the top using a combination of an infrared photodiode sensor (S132C, Thorlabs) and a power meter (PM100D, Thorlabs).

### 6.3 Band structure and band-edge mode simulations

All numerical calculations were performed using a three-dimensional FDTD simulation software package (ANSYS Lumerical FDTD, ANSYS). For photonic band structure calculations, the PhC unit cell constituted the simulation field, with Bloch boundary conditions applied. Mode profiles were extracted using a field-profile monitor. The effective refractive indices of the SCH-MQW layer and the InP cladding layers were assumed to be 3.4 and 3.17, respectively. The effective refractive index of the InP cladding layer with etched air holes was assumed to be the areal average of the refractive indices of InP and air.

## Supplementary Material

Supplementary Material Details
