# Supplementary material for: 1,550-nm photonic crystal surface-emitting laser diode fabricated by single deep air-hole etch
Source: Nanophotonics. 2025 Feb 14;14(4):515–22. doi: 10.1515/nanoph-2024-0760 (PMC11833739; doi:10.1515/nanoph-2024-0760)
Supplement: Supplementary file 1 — Supplementary Material Details [file j_nanoph-2024-0760_suppl_001.docx]

[Supplementary Information]

1550-nm photonic crystal surface-emitting laser diode fabricated by single deep air-hole etch

MYEONGEUN KIM^1,2^, YE-SEONG SONG^1,2^, LAKJONG JeoNG^1,2^, TAE-YUN LEE^1,2^, HYO SEOK CHOI^3^, IN KIM^3^, MYUNGJAE LEE^2,4,†^, AND HEONSU JEON^1,2,5,*^

^1^Department of Physics and Astronomy, Seoul National University, Seoul 08826, Republic of Korea

^2^Inter-university Semiconductor Research Centre, Seoul National University, Seoul 08826, Republic of Korea

^3^OE Solutions, Gwangju 61080, Republic of Korea

^4^Department of Materials Science and Engineering, Seoul National University, Seoul 08826, Republic of Korea

^5^Research Institute of Advanced Materials, Seoul National University, Seoul 08826, Republic of Korea

^6^Institute of Applied Physics, Seoul National University, Seoul 08826, Republic of Korea

†e-mail: myungjae@snu.ac.kr; *email: hsjeon@snu.ac.kr

List of Contents

[S1. InP-based epistructure for the PCSEL 3](#_Toc143857038)

[S2. Guided mode profiles in the vertical direction 4](#_Toc143857039)

[S3. Field profiles of the Γ_3_/Γ_4_ band-edge modes 5](#_Toc143857040)

[S4. Optical transmission through the thin-metal contact 6](#_Toc143857041)

S1. InP-based epistructure for the PCSEL


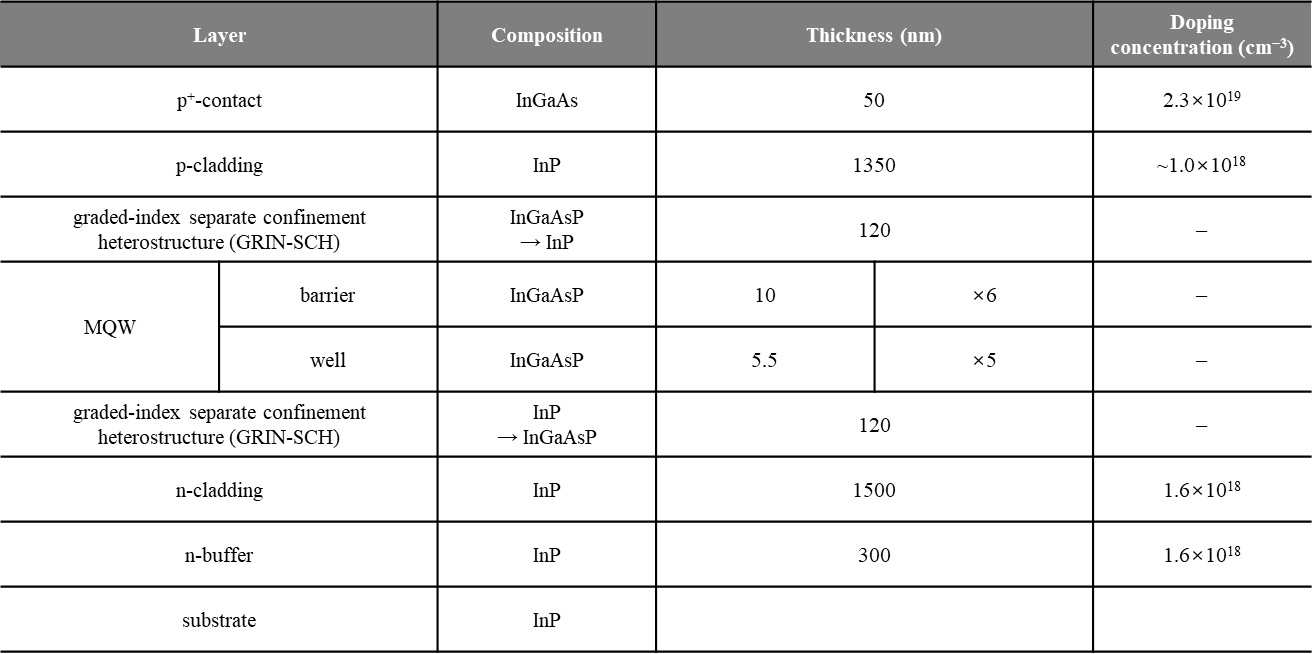


Fig. S1 | InP-based 1550-nm LD epistructure used for the fabrication of the proposed PCSEL. The epistructure is a typical Fabry-Pérot LD structure, containing five quantum wells in a graded-index separate confinement heterostructure (GRIN-SCH) configuration. For the purpose of demonstrating the flexibility in epistructure design, the electron blocking layer is intentionally excluded, which is normally embedded in the p-cladding layer in a standard LD epistructure. Please note that this epistructure information is provided to show its commonness, not its specialness, and also that any similar Fabry-Pérot LD structure should be equally employable for the fabrication of our PCSEL.

S2. Guided mode profiles in the vertical direction


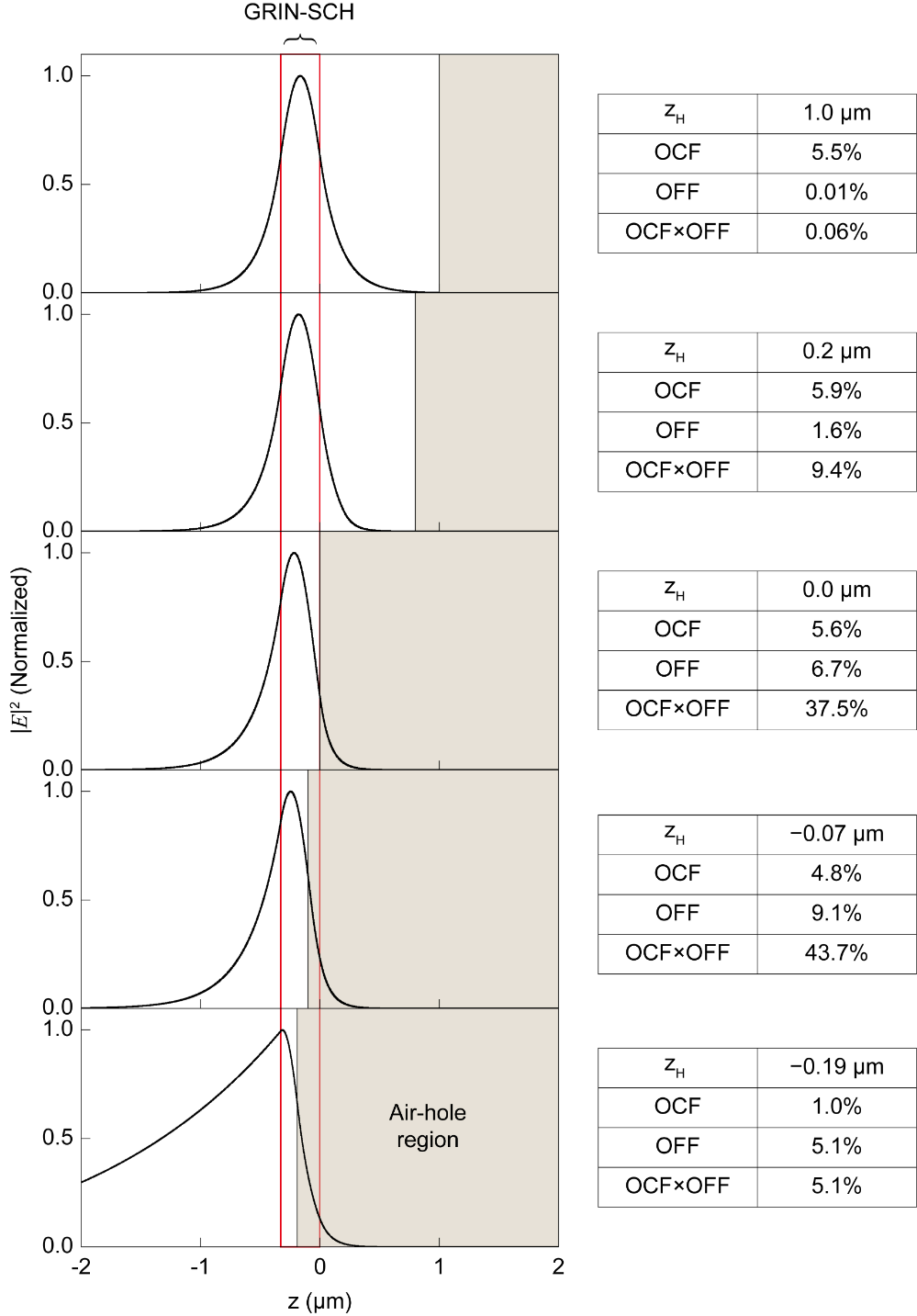


Fig. S2 | Vertical guided mode profiles. Vertical mode profiles are calculated and shown for a few representative air-hole depths: from top, *z*_H_ = 1.00, 0.20, 0, −0.07, and −0.19 μm in terms of the position of the air-hole bottom. In each plot, the shaded area indicates the section where the air holes are formed, while the two red lines are the boundaries between the InGaAsP GRIN-SCH and the InP-claddings, thus defining the InGaAsP GRIN-SCH region. The tables on the right summarize the OCF, OFF, and OCF×OFF, all deduced from the corresponding plots.

S3. Field profiles of the Γ_3_/Γ_4_ band-edge modes


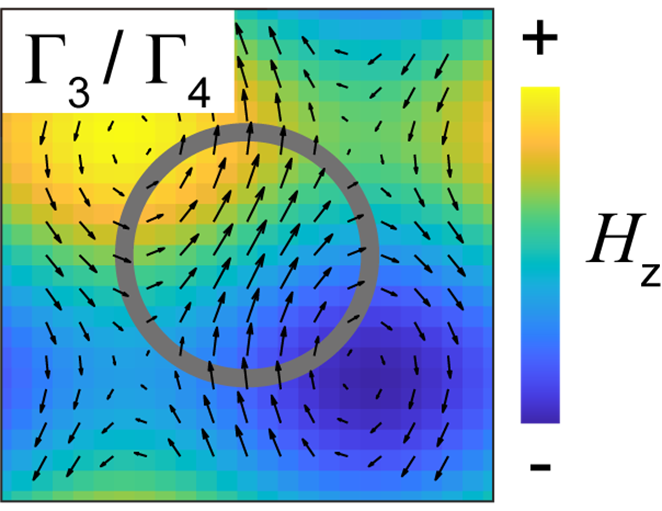


Fig. S3 | Electric and magnetic field profiles of the degenerate Γ_3_/Γ_4_ band-edge mode. Calculated electric (*E*_||_; arrows) and magnetic (*H*_z_; colors) field profiles are shown for the degenerate Γ_3_/Γ_4_ band-edge mode across a unit cell of the square-lattice PhC structure. The gray circle in the middle depicts the air hole. The large electric field strength within the air hole indicates that the mode belongs to an air mode, while the odd symmetry in the *H*_z_ field profile infers that the mode is a non-BIC mode characterized by low *Q*, thus inappropriate for lasing.

S4. Optical transmission through the thin-metal contact


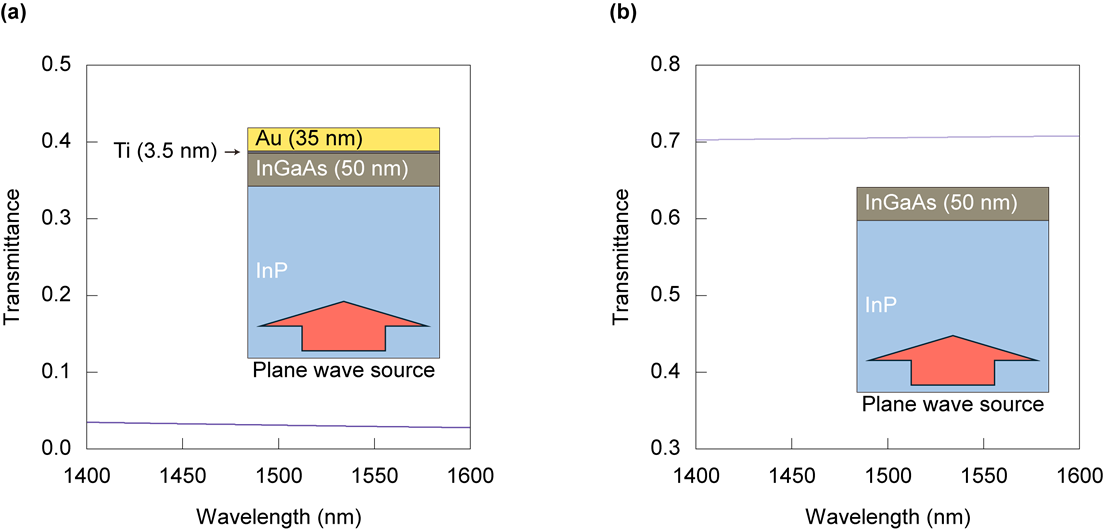


Fig. S4 | Optical transmittance spectrum through the thin-metal contact. a, Calculated optical transmittance spectrum through the thin-metal contact. The inset shows the structural configuration used in the simulation: a plane wave launched from inside the InP-cladding layer impinges on the p-contact composed of a 50-nm-thick InGaAs contact layer and the thin-metal contact (3.5-nm-thick Ti and 35-nm-thick Au). A book by E. D. Palik^1^ was consulted for the dispersive optical constants of Ti and Au. b, Optical transmittance spectrum calculated for the reference structure (for a purely comparison purpose) before the formation of metal contact, which is depicted in the inset.

--------------------------------------------------------------------------------------------------------------

^1^Handbook of Optical Constants of Solids I–III, E. D. Palik, Elsevier (1985).
